# Characterization of a novel non-specific nuclease from thermophilic bacteriophage GBSV1

**DOI:** 10.1186/1472-6750-8-43

**Published:** 2008-04-28

**Authors:** Qing Song, Xiaobo Zhang

**Affiliations:** 1 Key Laboratory of Marine Biogenetic Resources, Third Institute of Oceanography, State Oceanic Administration, Xiamen 361005, The People's Republic of China

## Abstract

**Background:**

Thermostable enzymes from thermophiles have attracted extensive studies. In this investigation, a nuclease-encoding gene (designated as *GBSV1-NSN*) was obtained from a thermophilic bacteriophage GBSV1 for the first time.

**Results:**

After recombinant expression in *Escherichia coli*, the purified GBSV1-NSN exhibited non-specific nuclease activity, being able to degrade various nucleic acids, including RNA, single-stranded DNA and double-stranded DNA that was circular or linear. Based on sequence analysis, the nuclease shared no homology with any known nucleases, suggesting that it was a novel nuclease. The characterization of the recombinant GBSV1-NSN showed that its optimal temperature and pH were 60°C and 7.5, respectively. The results indicated that the enzymatic activity was inhibited by enzyme inhibitors or detergents, such as ethylene diamine tetraacetic acid, citrate, dithiothreitol, β-mercaptoethanol, guanidine hydrochloride, urea and SDS. In contrast, the nuclease activity was enhanced by TritonX-100, Tween-20 or chaps to approximately 124.5% – 141.6%. The *K*_m _of GBSV1-NSN nuclease was 231, 61 and 92 μM, while its *k*_cat _was 1278, 241 and 300 s^-1 ^for the cleavage of dsDNA, ssDNA and RNA, respectively.

**Conclusion:**

Our study, therefore, presented a novel thermostable non-specific nuclease from thermophilic bacteriophage and its overexpression and purification for scientific research and applications.

## Background

Nucleases are defined as a group of enzymes which are capable of hydrolyzing the phosphodiester linkages of nucleic acids. According to the substrates they hydrolyze, nucleases are divided into two groups: sugar specific nucleases (deoxyribonucleases and ribonucleases) and sugar non-specific nucleases [1]. Sugar non-specific nucleases, characterized by their ability to hydrolyze both DNA and RNA without exhibiting pronounced base preferences, have been detected from a wide variety of sources, such as virus, bacteria, fungi and animals [1-4]. Many of them are extracellular, but some have been found in nuclei, vacuoles, mycelia, mitochondria, conidia, microplasmodia and periplasm [5-11]. Sugar non-specific nucleases play very important roles in different aspects of basic genetic mechanisms, including their participation in mutation avoidance, DNA repair, DNA replication and recombination, scavenging of nucleotides and phosphates for the growth and metabolism, host defense against foreign nucleic acid molecules, programmed cell death and establishment of an infection. Due to their important roles in nucleic acid metabolisms, the sugar non-specific nucleases have been extensively used in molecular biology researches, for example the determination of nucleic acid structure, the rapid sequencing of RNA, the removal of nucleic acids during protein purification and the use as antiviral agents [1,12-14].

Up to date, more than 30 nucleases have been obtained from microorganisms, such as *staphylococcal *nuclease from *Staphylococcus aureus, S. marcescens *nuclease from *Serratia marcescens*, S1 nuclease from *Aspergillus oryzae*, P1 nuclease from *Penicillium citrinum*, BAL31 nuclease from *Alteromonas espejiana *and NucA from *Anabaena *[15-17]. With regard to virus, only the PC1 protein from Fowlpox virus and rWSSV-NSN from shrimp white spot syndrome virus are identified as non-specific nucleases [2]. However, all the non-specific nucleases are obtained from mesophiles or mesophilic viruses. No study has reported on nucleases from thermophilic viruses. Compared with mesophilic enzymes, thermostable nucleases may possess novel properties in structures and biological functions.

In the present study, a non-specific nuclease gene (termed as *GBSV1-NSN*) was cloned from a thermophilic bacteriophage GBSV1 for the first time. The sequence homology analysis revealed that it was a novel nuclease. The recombinant GBSV1-NSN nuclease was further characterized. It was shown that the GBSV1-NSN enzyme was thermostable.

## Results

### Recombinant expression and determination of a novel non-specific nuclease GBSV1-NSN

**Figure 1:**
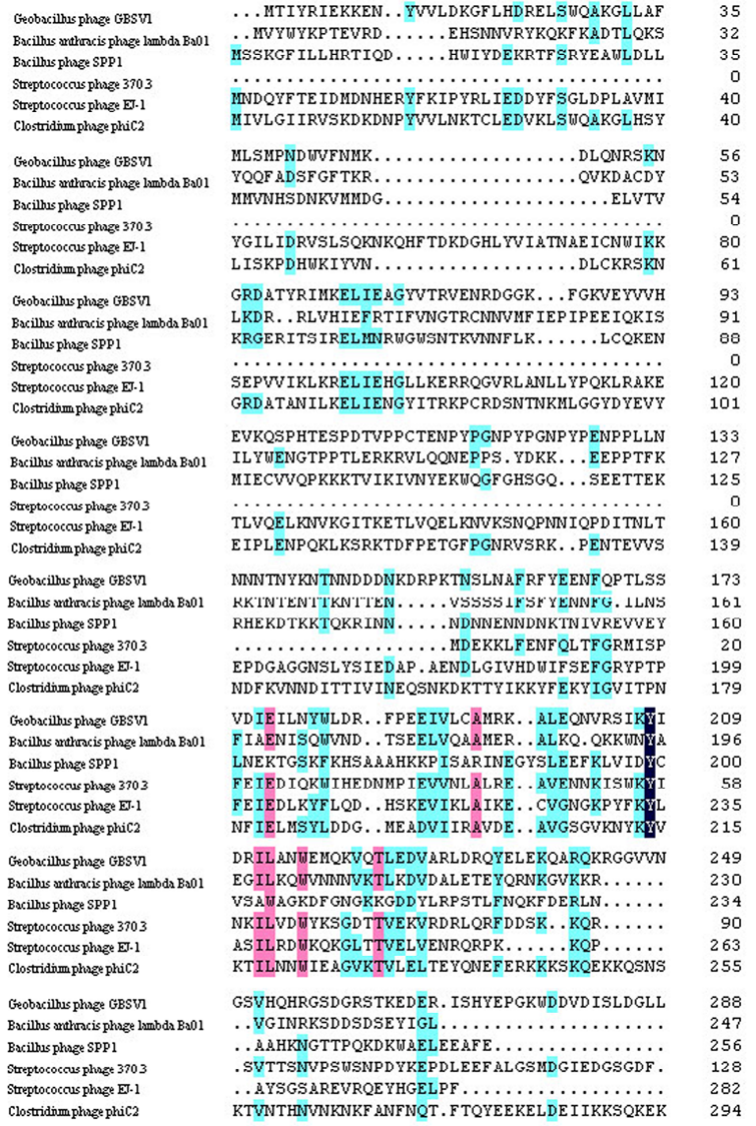
**Multiple alignments of phage proteins that contained DnaD-like domains**. Phage proteins containing DnaD-like domain were from *Geobacillus *phage GBSV1 [GenBank: ABK63794], *Bacillus anthracis *phage lambda Ba01 [GenBank: AAP24466], *Bacillus *phage SPP1 [GenBank: NP_690731], *Streptococcus *phage 370.3 [GenBank: AAK33864], *Streptococcus *phage EJ-1 [GenBank: NP_945250] and *Clostridium *phage phiC2 [GenBank: YP_001110781]. The DnaD-like domain of GBSV1-NSN held positions 158–227.

**Figure 2:**
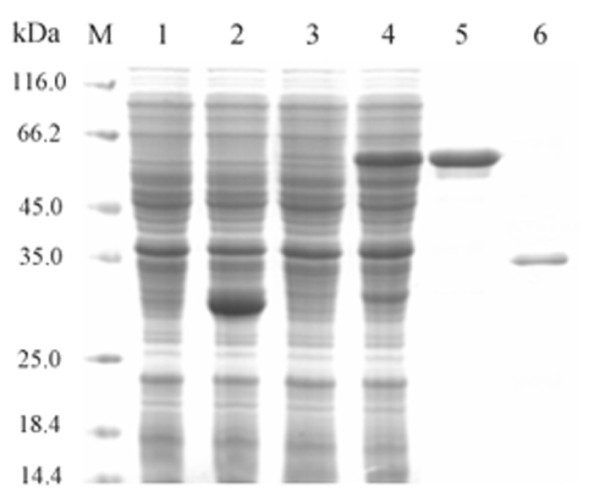
**SDS-PAGE of expressed and purified protein encoded by the *GBSV1-NSN *gene**. Lanes M, protein marker; 1, control (vector only), non-induced; 2, control (vector only), induced; 3, recombinant bacterium (containing *GBSV1-NSN *gene), non-induced; 4, recombinant bacterium (containing *GBSV1-NSN *gene), induced; 5, purified GST-GBSV1-NSN fusion protein; 6, purified GBSV1-NSN.

Based on genome sequence analysis of thermophilic bacteriophage GBSV1, an open reading frame (ORF) [GenBank: EF079892] of GBSV1 shared homologies with phage replication proteins (Fig. 1), which contained a DnaD-like domain, suggesting that the protein encoded by this ORF had the DNA binding capacity. In order to identify its biological function, the ORF (termed as *GBSV1-NSN *gene) was expressed as GST fusion protein in *E*.*coli*. After induction with IPTG at 37°C, the induced and non-induced recombinant bacterium (containing *GBSV1-NSN *gene) and control bacterium (vector only) were analyzed by SDS-PAGE. A band (about 59.8 kDa) corresponding to the GST-GBSV1-NSN fusion protein was observed in the induced recombinant bacterium containing *GBSV1-NSN *gene (Fig. 2, lane 4), while no protein was found in the same positions in the induced and non-induced controls (vector only), showing that the *GBSV1-NSN *gene was expressed. After purification by affinity chromatography, a GST-GBSV1-NSN fusion protein was obtained (Fig. 2, lane 5). The fusion protein GST-GBSV1-NSN was efficiently cleaved by thrombin, yielding the purified GBSV1-NSN with a molecular mass of 33.8 kDa (Fig. 2, lane 6).

**Figure 3:**
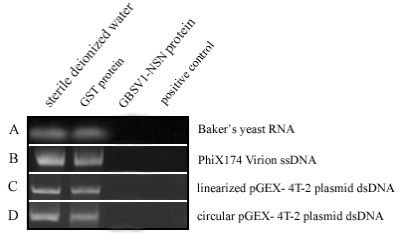
**Digestion of various nucleic acids by the purified GBSV1-NSN protein**. The purified GST protein was used as negative control. The commercial RNase A (A) or DNase I (B, C and D) was also included as positive control. The protein solutions were shown on the top and the nucleic acids were indicated on the right. 1 μg of nucleic acids were respectively incubated with 1.5 μg of the purified GBSV1-NSN protein in 20 μl of reaction buffer at 37°C for six hours.

In an attempt to determine the DNA binding capacity of the recombinant GBSV1-NSN, the purified GBSV1-NSN protein was incubated with the genomic DNA of bacteriophage GBSV1. Surprisingly, the DNA was degraded by the GBSV1-NSN protein, suggesting it might be a nuclease. To evaluate its substrate specificity, the purified GBSV1-NSN protein was incubated with various nucleic acids including the circular pGEX-4T-2 plasmid dsDNA, the linear pGEX-4T-2 plasmid dsDNA, the PhiX174 virion ssDNA and the baker's yeast RNA. The results showed that all the nucleic acids could be degraded by the GBSV1-NSN protein (Fig. 3), indicating that it was a non-specific nuclease.

Homology analysis revealed that the GBSV1-NSN protein shared low identities with known nucleases. It had 10.0%, 13.3%, 13.5%, 10.9% and 12.1% identities with those of *Staphylococcus aureus *thermonuclease [GenBank: P00644], *Serratia marcescens *nuclease [GenBank: P13717], *Anabaena *sp. PCC 7120 nuclease [GenBank: YP_227663], *Homo sapiens *EndoG [GenBank: Q14249] and WSSV-NSN nuclease [GenBank: AAW88443], respectively. These data suggested that the GBSV1-NSN was a novel non-specific nuclease.

### The effects of temperature and pH on GBSV1-NSN enzymatic activity

**Figure 4:**
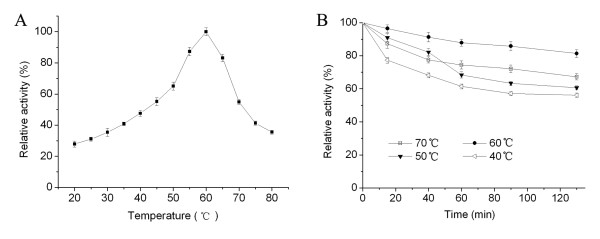
**Optimum temperature (A) and thermostability (B) of the GBSV1-NSN nuclease**. The nuclease activity of GBSV1-NSN was assayed at various temperatures ranging from 20°C to 80°C. To evaluate the thermostability, the residual enzymatic activities were monitored after incubation of the enzyme solutions (295 μg/ml) in the absence of herring dsDNA (100 μg/ml) at 40, 50, 60 or 70°C (pH 7.5) for 15, 40, 60, 90 or 130 min. The nuclease activity assay was conducted using 100 μg/ml of herring dsDNA as substrate. Each point represented the mean of triplicate assays and the error bars showed the standard deviations.

The nuclease activity estimated at different temperatures indicated that the optimum temperature for the recombinant GBSV1-NSN was 60°C (Fig. 4A). The GBSV1-NSN nuclease activity was decreased with higher temperatures above the optimum. Thermostability assays showed that the GBSV1-NSN nuclease was most stable at 60°C (pH 7.5), retaining more than 80% of its enzymatic activity for at least 2 hours (Fig. 4B).

**Figure 5:**
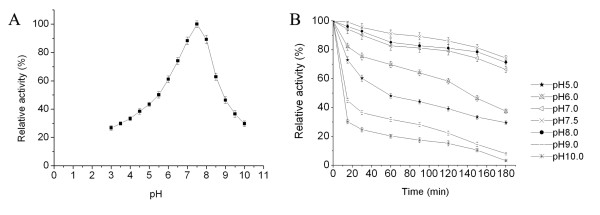
**Effects of pH on enzymatic activity (A) and stability (B)**. The optimum pH was assayed after incubation in various pH buffers (pH 3.0 to 10.0) at 60°C for 15 min. For pH stability experiments, the GBSV1-NSN was pre-incubated in a series of pH buffers (pH 5.0 to 10.0) for 15, 30, 60, 90, 120, 150 or 180 min before assay. Each reaction mixture consisted of 100 μg/ml herring dsDNA and 295 μg/ml of the purified GBSV1-NSN protein. Each point represented the mean of triplicate assays and the error bars indicated the standard deviations.

Based on the effects of pH on enzymatic activity, the GBSV1-NSN nuclease exhibited optimal activity at pH 7.5 (Fig. 5A). The recombinant nuclease was stable in the neutral pH ranging from 7.0 to 8.0 (Fig. 5B). It presented higher stability after incubation at 60°C for 3 hours, showing 66, 74 and 71 % residual activity at pH 7.0, 7.5 and 8.0, respectively (Fig. 5B).

### Responses of the GBSV1-NSN nuclease to metal ions, enzyme inhibitors and detergents

The effects of different metal ions on the GBSV1-NSN nuclease activity were evaluated as shown in Table 1. In the absence of metal ion, the GBSV1-NSN nuclease was active. The enzymatic activity was obviously stimulated in the presence of Mn^2+ ^and Zn^2+ ^at 10 mM concentration each. Metal ions Li^+^, Na^+^, K^+^, Cs^+^, Ca^2+^, Mg^2+^, Ni^2+^, Sr^2+ ^and Ba^2+ ^at different concentrations showed very slight inhibition of the GBSV1-NSN activity, retaining more than 80% of its initial activity. However, its activity was strongly inhibited by Cu^2+ ^and Fe^3+^. With the increasing in Cu^2+ ^or Fe^3+ ^concentration, the nuclease activity decreased sharply.

**Table 1:** Effects of metal ions on the GBSV1-NSN enzymatic activity. The concentrations of metal ions represented their concentrations in the incubation solutions with the nuclease and in the final nuclease reactions. The error limits shown were the standard deviations.

Metal ions	Relative activity (%)		
Concentrations in incubation solution/in final nuclease reaction	0.1 mM/0.05 mM	1 mM/0.5 mM	10 mM/5 mM

None	100	100	100
Mn^2+^	92.4 ± 2.9	102.5 ± 3.1	130.8 ± 4.1
Zn^2+^	101.4 ± 3.1	114.7 ± 3.0	118.4 ± 2.9
Sr^2+^	96.8 ± 2.8	101.1 ± 2.9	102.3 ± 2.1
Ba^2+^	98.0 ± 3.1	97.8 ± 2.8	92.4 ± 2.9
Mg^2+^	93.3 ± 1.9	92.9 ± 2.2	87.8 ± 2.6
Ca^2+^	98.3 ± 2.2	94.6 ± 2.4	92.2 ± 2.8
Ni^2+^	93.2 ± 1.0	89.5 ± 1.5	86.4 ± 2.2
Li^+^	93.6 ± 3.0	92.3 ± 2.8	91.0 ± 2.4
Na^+^	92.5 ± 2.9	81.9 ± 2.7	80.8 ± 1.7
K^+^	95.1 ± 1.9	87.5 ± 2.1	83.9 ± 2.6
Cs^+^	98.7 ± 3.2	95.1 ± 1.6	89.8 ± 2.5
Cu^2+^	74.2 ± 2.3	55.5 ± 1.4	3.6 ± 0.1
Fe^3+^	67.7 ± 1.5	22.3 ± 0.8	0.7 ± 0.1

The results indicated that the recombinant nuclease was active when some metal chelators, thiol reagents and detergents were used (Table 2). However, the enzymatic activity was reduced by some enzyme inhibitors or detergents, such as ethylene diamine tetraacetic acid (EDTA), citrate, dithiothreitol (DTT), β-mecaptoethanol (2-ME), guanidine hydrochloride, urea and SDS. Among them, 10 mM of DTT and 0.6 M of guanidine hydrochloride led to a significant reduction of enzyme activity by 34.1% and 51.0%, respectively. In contrast, the nuclease activity was enhanced by TritonX-100, Tween-20 or chaps to approximately 124.5 – 141.6%.

**Table 2:** Effects of metal chelators, thiol reagents and detergents on the GBSV1-NSN nuclease activity. The concentrations of additives represented their concentrations in the incubation solutions with the nuclease and in the final nuclease reactions. The error limits shown were the standard deviations.

Reagents	Concentrations in incubation solution/in final nuclease reaction	Relative activity (%)
None	0	100
EDTA	1 mM/0.5 mM	96.9 ± 3.2
	10 mM/5 mM	85.1 ± 2.8
Citrate	1 mM/0.5 mM	94.2 ± 2.9
	10 mM/5 mM	66.8 ± 2.2
DTT	1 mM/0.5 mM	52.8 ± 1.6
	10 mM/5 mM	34.1 ± 1.2
β-ME	1 mM/0.5 mM	89.5 ± 2.5
	10 mM/5 mM	87.8 ± 2.7
Urea	1 M/0.5 M	63.9 ± 2.1
Guanidine hydrochloride	0.6 M/0.3 M	51.0 ± 1.8
SDS	0.1 %/0.05%	61.8 ± 2.0
	1 %/0.5%	73.4 ± 2.2
Tween-20	0.1 %/0.05%	138.9 ± 3.7
	1 %/0.5%	141.6 ± 3.9
TritonX-100	0.1 %/0.05%	124.5 ± 3.2
	1 %/0.5%	133.7 ± 3.6
Chaps	0.1 %/0.05%	126.2 ± 2.9
	1 %/0.5%	140.6 ± 3.2

### Kinetic parameters of the GBSV1-NSN nuclease

**Figure 6:**
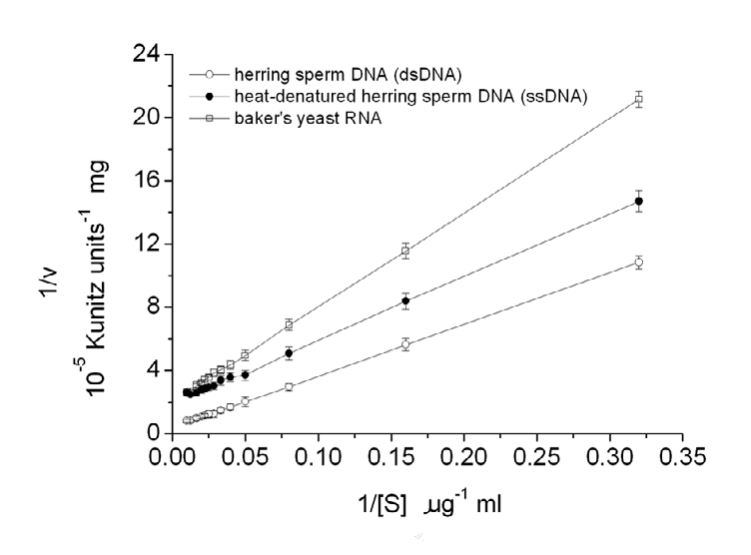
**The Lineweaver-Burke plot**. The kinetic parameters *K*_m _and *V*_max _were estimated by linear regression from Lineweaver-Burk plots. The nucleic acid concentrations were varied in the range of 0–100 μg/ml. All nuclease activity assays were conducted at 60°C and pH 7.5. Each point represented the mean of triplicate assays and the error bars represented the standard deviations.

The kinetic parameters of the nuclease were obtained from Lineweaver-Burke plot of specific activities at 60°C with different substrates including the double-stranded DNA (dsDNA), single-stranded DNA (ssDNA) and RNA. The results revealed that its *K*_m _value was 75, 20, 30 μg/ml and *V*_max _value was 2.5 × 10^5^, 5 × 10^4^, 5 × 10^4 ^Kunitz units·mg^-1 ^for the dsDNA, ssDNA and RNA, respectively (Fig. 6). After calculations, the *k*_cat _of GBSV1-NSN nuclease was estimated to be 1278, 241 and 300 s^-1 ^for the dsDNA, ssDNA and RNA substrates, respectively (Fig. 6).

## Discussion

Driven by increasing industrial demands for thermostable biocatalysts, thermophiles have attracted more and more attention as a valuable source of novel enzymes [18]. Most thermophilic enzymes were extensively studied from thermophilic archaea and bacteria. However only a few enzymes from thermophilic bacteriophages have been characterized, for example thermostable RNA ligase [19], polynucleotide kinase [20] and lytic enzyme [21]. Therefore, thermophilic bacteriophages are a considerable resource of thermophilic enzymes. In this investigation, the recombinant GBSV1-NSN protein from the thermophilic bacteriophage GBSV1 exhibited non-specific nuclease activity.

The results showed that the GBSV1-NSN nuclease was active at temperatures ranging from 20°C to 80°C with an optimal temperature of 60°C, which was higher than those of most of the reported non-specific nucleases. In the respect of effects of metal ions on the enzymatic activity, the GBSV1-NSN was similar to the nuclease α from *U. maydis *and wheat chloroplast nuclease that did not show an obligate requirement of metal ions for their activities [22]. It was found that Mn^2+ ^or Zn^2+ ^could stimulate the GBSV1-NSN activity. This stimulation was also observed in the ColE7 nuclease which bound a transition metal ion Mn^2+ ^or Zn^2+ ^in its H-N-H motif [23]. However EDTA had a mild effect in reducing the activity of the GBSV1-NSN enzyme. The data suggested that metal ions had a stimulatory effect but were not obligatory for nuclease activity and that they might play roles in stabilizing the enzymatic structure. It could not be ruled out whether the GBSV1-NSN enzyme was copurified with some tightly bound metal ions, as was the case of some other nucleases. This point remained to be further addressed. The amino acid sequence analysis of the GBSV1-NSN revealed the existence of two Cys residues, indicating that the nuclease might contain a disulfide bridge. It was found that the GBSV1-NSN nuclease activity was inhibited by thiol reagents DTT or β-ME, suggesting that its disulfide bridge could play an important role in determining the activity of this nuclease. However the enzyme was stimulated by TritonX-100, Tween-20 and chaps. Comparisons of the catalytic properties of the GBSV1-NSN with the known nucleases suggested that it had a similar efficiency in dsDNA hydrolysis as the *Serratia *nuclease (SMNase) [24] and *F. succinogenes *DNase A [3]. However, it had lower catalytic efficiency than that of *Anabaena *NucA in cleavage of various nucleic acids and than that of *Staphylococcus *nuclease (SNase) in cleavage of ssDNA (Table 3).

**Table 3:** Steady-state kinetic parameters for the cleavage of various nucleic acids by GBSV1-NSN and other nucleases.

Enzyme	Substrate	*K*_m_		*k*_cat_	*k *_cat_ / *K*_m_	Reference
		ug·ml^-1^	μM	s^-1^	uM^-1^·s^-1^	
GBSV1 -NSN	herring sperm DNA	75	231	1278	5.5	this work
	heat-denatured DNA	20	61	241	4.0	this work
	Baker's yeast RNA	30	92	300	3.3	this work
NucA	herring sperm DNA	53	164	1921	11.7	[24]
	scDNA	82	253	2111	8.3	[24]
	*E. coli *tRNA^Leu^	83	257	2255	8.7	[24]
	heat-denatured DNA	41	126	2170	17.2	[24]
SNase	heat-denatured DNA	15	46	765	16.6	[24]
SMNase	herring sperm DNA	20	61	980	16.1	[24]
DNase A	herring sperm DNA	20	61	330	5.4	[3]

Although the GBSV1-NSN protein displayed non-specific nuclease activity, it shared no homology to any known nucleases in the primary amino acid sequences. Our data indicated that the GBSV1-NSN was a novel member of non-specific nuclease family, which was thermostable and disulfide bridge-dependent.

Based on sequence analysis, the GBSV1-NSN protein was highly homologous with the phage replication protein and contained a DnaD-like domain, suggesting that it might play important roles in bacteriophagic DNA replication. As well known, DNA replication is divided into three main stages, that is initiation, elongation and termination. In some gram-positive bacteria with low G+C content, the DNA replication initiation involves remodeling of a replication origin through the action of the main initiator protein DnaA and primosomal multiprotein cascades including DnaD. These initiator proteins interact with helicases to recruit the DNA primases. DnaD is believed to be involved in the cascade for helicase recruitment [25]. Considering that the GBSV1-NSN nuclease contained DnaD domain, this protein might possess multiple biochemical activities, including the nuclease activity and involvements in the DNA replication initiation, as well as in mismatch bacteriophagic DNA repair.

Up to date, the non-specific nucleases already available are obtained from mesophiles. The discovery of thermostable GBSV1-NSN nuclease in this study would facilitate the applications of nucleases in molecular biology researches and in industry at high temperatures, such as the determination of nucleic acid structure, the removal of nucleic acids during protein purification and the use as antiviral agents. Those are merited to be further studied.

## Conclusion

In this study, we have determined a novel thermostable non-specific nuclease from thermophilic bacteriophage for the first time, which shared no homology with any known nucleases. The characterizations of the recombinant GBSV1-NSN showed that it was a thermostable nuclease. In this investigation, the recombinant GBSV1-NSN could be obtained in large quantity by expression in *E. coli*. This would facilitate its biotechnological applications.

## Methods

### Thermophilic bacteriophage GBSV1

During the culture of *Geobacillus *sp. 6K51, phage plaques were observed from a thermophilic bacterium isolated from an offshore hot spring in Xiamen of China. The thermophilic bacteriophage (termed as GBSV1) was purified from its host, which was grown at 60°C with shaking (150 rpm) in the medium consisting of 0.2% NaCl, 0.4% yeast extract and 0.8% tryptone (pH 7.0). The GBSV1 contained a double linear genomic DNA of 34683 bp, which had the capacity encoding 55 open reading frames (ORFs). The genome sequencing revealed that it was a novel bacteriophage.

### Cloning and recombinant expression and purification of GBSV1-NSN protein

The sequence analysis of GBSV1 genome revealed an ORF sharing high homologies with phage replication proteins. To characterize the protein encoded by this ORF (termed as *GBSV1-NSN *gene), it was expressed in *Escherichia coli *BL21 (DE3) as a fusion protein with glutathione *S*-transferase (GST). The *GBSV1-NSN *gene was amplified from the GBSV1 genomic DNA using primers 5'-*CGGGATCC*ATGACGATATATCGCATCG-3' (BamHI site, italic) and 5'-*GCGAATTC*TTATAGAAGTCCATCGAGG-3' (EcoRI site, italic). The amplified fragment was cloned into pGEX-4T-2 downstream of GST. The recombinant bacteria were induced with 1 mM of isopropyl-β-D-thiogalactopyranoside (IPTG) when OD_600 _reached 0.6–0.8. After incubation for an additional 4 to 5 h at 37°C, cultures were harvested by centrifugation (4,000 × *g*, 10 min, 4°C) and resuspended in 20 ml of ice-cold phosphate-buffered saline (PBS, pH 7.4). Cells were lysed by sonication and the cell debris was removed by centrifugation at 10,000 × *g *for 15 min. The supernatants were mixed gently with 1 ml glutathione- sepharose beads (Sigma, America) for 2 h at 4°C. After rinsing with ice-cold PBS, the GST fusion protein was pooled with a reducing buffer of 50 mM Tris-HCl and 10 mM reduced glutathione\(pH 8.0). The purified fusion protein was incubated with thrombin (Sigma, America) in a reaction buffer of 50 mM Tris-HCl, 150 mM NaCl, 2.5 mM CaCl_2 _and 0.1% β-mecaptoethanol\(pH 8.0) at 22°C for 8 h. Subsequently it was applied to glutathione-agarose beads (Sigma, USA) to remove GST. The purified protein was dialyzed in 50 mM Tris-HCl (pH8.0).

Homology searches were performed against the sequences in the GenBank database using the BLAST program. Alignments of deduced amino acid sequence were generated with DNAMAN program.

### Substrates for the recombinant GBSV1-NSN nuclease

To determine the specificity of substrate for the recombinant GBSV1-NSN nuclease, 1 μg of the circular pGEX-4T-2 plasmid dsDNA, the linear pGEX-4T-2 plasmid dsDNA, the PhiX174 virion ssDNA (New England Biolabs, UK) or baker's yeast RNA (Sigma-Aldrich, USA) were respectively incubated with 1.5 μg of the purified GBSV1-NSN protein in 20 μl of a reaction buffer (20 mM Hepes, 50 mM KCl, 2 mM MgCl_2_, 0.2 mM EDTA, 0.1 mg/ml BSA and 10% glycerol, pH 7.5) at 37°C. Six hours later, 10 μl of the reaction solution was analyzed by electrophoresis on a 1.0 % agarose gel. In the assays, 1.5 μg of commercial DNase I (TaKaRa, Japan) and RNase A (Sigma-Aldrich, USA) were included as controls. All assays were conducted in triplicate.

### Nuclease activity assay of the recombinant GBSV1-NSN protein

The spectrophotometric method was used to measure the nuclease activity according to the protocol as described previously [3]. One Kunitz unit was defined as the amount of enzyme needed to cause an increase of absorbance of 0.001 ⊿*A*_260_·ml^-1^·min^-1^·cm^-1 ^at 60°C. Each reaction mixture consisted of 100 μg/ml RNA or DNA, 20 mM Tris-HCl (pH 7.5) and the recombinant GBSV1-NSN nuclease. After incubation for 15 min at 60°C, the reactions were stopped by equal volume of 5% ice-cold perchloric acid. The solutions were held on ice for 10 min and then centrifuged for 5 min at 8000 × g. The supernatants were measured using a Thermo Spectronic UNICAM UV 300 spectrophotometer set at 260 nm.

### Temperature optimum and thermostability of the GBSV1-NSN nuclease

The optimal temperature of the GBSV1-NSN nuclease was measured by performing the enzymatic activity assay at temperatures ranging from 20 to 80°C in the presence of herring sperm DNA (Sigma-Aldrich, USA) as substrate. The thermostability of nuclease was investigated at different temperatures 40, 50, 60 and 70°C after incubation of the enzyme solutions (295 μg/ml) in the absence of 100 μg/ml herring dsDNA (20 mM Tris-HCl, pH 7.5) for 15, 40, 60, 90 or 130 min, respectively. Residual activities were determined under nuclease activity assay conditions using 100 μg/ml of herring dsDNA as substrate. All assays were repeated for three times.

### pH optimum and pH-dependent stability of GBSV1-NSN

The effect of pH on nuclease activity was evaluated at the optimal temperature over a pH range of 3.0–10.0, using five different buffers (all at 20 mM concentrations): sodium formiate (pH 3.0, 3.5), sodium acetate (pH 3.5 to 6.0), imidazole (pH 6.0 to 7.0), Tris/HCl (pH 7.0 to 9.0) and ethanolamine (9.0 to 10.0), under nuclease activity assay conditions. Further study on the pH stability of nuclease was carried out at 60°C by pre-incubation of the enzyme solutions in Tris/HCl buffer systems (pH 5.0 to 10.0) in the absence of substrate for 15, 30, 60, 90, 120, 150 and 180 min, respectively. Then they were subjected to the nuclease activity assay. The assays were conducted in triplicate.

### Effects of metal ions on nuclease activity

The effects of metal ions on nuclease activity were determined by measuring the residual activity of GBSV1-NSN after separate incubation of the enzyme solutions with various metal ions at 60°C and pH 7.5 for 10 min. The concentrations of metal ions in the incubation solutions with the nuclease and in the final nuclease reactions were 0.1, 1.0 or 10 mM and 0.05, 0.5 or 5 mM, respectively. The assays were carried out for three times.

### Effects of metal chelators, thiol reagents and detergents on GBSV1-NSN activity

To determine the effects of metal chelators, the nuclease was pre-incubated with ethylene diamine tetraacetic acid (EDTA) or citrate at 60°C for 10 min, followed by the nuclease activity assay. The effects of thiol reagents and detergents on the nuclease activity were also monitored by the same procedure as described above. The concentrations of EDTA, citrate, dithiothreitol (DTT) and β-mecaptoethanol (2-ME) were 1 mM or 10 mM in the incubation solution with the nuclease and 0.5 mM or 5 mM in the final nuclease reaction, respectively. The concentrations of SDS, TritonX-100, Tween-20 or 3- [(3-cholamidopropyl) dimethylammonio]-1-propane sulfonate (chaps) were 0.1% or 1% (w/v) in the incubation solution with the nuclease and 0.05% or 0.5% in the final nuclease reaction, respectively. The concentrations of guanidine hydrochloride and urea were 0.6 M and 1 M in the incubation solution with the nuclease and 0.3 M and 0.5 M in the final nuclease reaction, respectively. All assays were performed in triplicate.

### Kinetic parameters of GBSV1-NSN

The protein concentration of purified GBSV1-NSN was determined based on the dye-binding assay method of Bradford [26], with bovine serum albumin (Sigma, USA) as a standard.

Steady-state kinetic parameters for the GBSV1-NSN nuclease were determined using the Kunitz assay with herring sperm DNA (dsDNA), heat-denatured herring sperm DNA (ssDNA) and baker's yeast RNA. In order to determine *K*_m _and *V*_max_, the nucleic acid concentrations were varied in the range of 0–100 μg·ml^-1^. All nuclease activity assays were conducted at 60°C in a Tris/HCl buffer (20 mM, pH 7.5). Typical Lineweaver-Burk plots were obtained when 1/[v] was plotted against 1/[S] [27]. Kinetic parameters were estimated by linear regression from Lineweaver-Burk plots. All assays were conducted for three times.

To compare the *K*_m _and *k*_cat _of GBSV1-NSN with those of the known nucleases, the *K*_m _values were converted from μg·ml^-1 ^to μM by using a mean molar mass of 330 g·mol ^-1^·nt ^-1^[28]. The *V*_max _values were converted to *k*_cat _by assuming that the molar mass per nucleotide was 330 g·mol ^-1 ^and that a change in absorbance of 0.3 *A*_260 _accompanied the degradation of 50 μg·ml^-1^ DNA and 0.25 *A*_260 _accompanied the degradation of 50 μg·ml^-1 ^RNA [24].

### Statistical analysis

All the numerical data were analyzed by one-way ANOVA and expressed as mean ± standard deviation.

## Authors' contributions

QS conducted the experiments and analyzed the data. XZ drafted the assays, participated in data analysis and wrote the manuscript. All authors read and approved the final manuscript.

## References

[B1] Mishra NC (2002). Nucleases: Molecular Biology and Applications.

[B2] Li L, Lin S, Yang F (2005). Functional identification of the non-specific nuclease from white spot syndrome virus. Virology.

[B3] Maclellan SR, Forsberg CW (2001). Properties of the major non-specific endonuclease from the strict anaerobe *Fibrobacter succinogenes *and evidence for disulfide bond formation in vivo. Microbiology.

[B4] Samejima K, Earnshaw WC (2005). Trashing the genome: the role of nucleases during apoptosis. Nat Rev Mol Cell Biol.

[B5] Ikeda S, Kawasaki N (2001). Isolation and characterization of the *Schizosaccharomyces *pombe cDNA encoding the mitochondrial endonuclease. Biochim Biophys Acta.

[B6] Bouex P, Sabourin M, Chaignepain S, Castroviejo M, Laquel-Robert P (2002). Purification and characterization of an endo-exonuclease from *Podospora anserine *mitochondria. Biochim Biophys Acta.

[B7] Wu SI, Lo SK, Shao CP, Tsai HW, Hor LI (2001). Cloning and characterization of a periplasmic nuclease of *Vibrio vulnificus *and its role in preventing uptake of foreign DNA. Appl Environ Microbiol.

[B8] Sopwith WF, Debrabant A, Yamage M, Dwyer DM, Bates PA (2002). Developmentally regulated expression of a cell surface class I nuclease in *Leishmania mexicana*. Int J Parasitol.

[B9] Yamage M, Debrabant A, Dwyer DM (2000). Molecular characterization of hyperinducible, surface membrane-anchored, class I nuclease of trypanosomatid parasite. J Biol Chem.

[B10] Accetto T, Avgustin G (2001). Non-specific DNAases from the rumen bacterium *Prevotella bryantii*. Folia Microbiol.

[B11] Al-Khaldi SF, Durocher LL, Martin SA (2000). Deoxyribonuclease activity in *Selenomonas ruminantium*, *Streptococcus bovis*, and *Bacteriodes ovatus*. Curr Microbiol.

[B12] Desai NA, Shankae V (2003). Single-strand-specific nucleases. FEMS Microbiol Rev.

[B13] Rangarajan ES, Shankar V (2001). Sugar non-specific endonucleases. FEMS Microbiol Rev.

[B14] Hsia KC, Li CL, Yuan HS (2005). Structural and functional insight into sugar-nonspecific nucleases in host defense. Curr Opin Struct Biol.

[B15] Meiss G, Gimadutdinow O, Haberland B, Pingoud A (2000). Mechanism of DNA cleavage by the DNA/RNA-non-specific *Anabaena *sp. PCC 7120 endonuclease NucA and its inhibition by NuiA.. J Mol Biol.

[B16] Korn C, Meiss G, Gast F, Gimadutdinow O, Urbanke C, Pingoud A (2000). Genetic engineering of *Escherichia coli *to produce a 1:1 complex of the *Anabaena *sp. PCC 7120 nuclease NucA and its inhibitor NuiA. Gene.

[B17] Ghosh M, Meiss G, Pingoud A, London RE, Pedersen LC (2005). Structural insights into the mechanism of nuclease A, a betabeta alpha metal nuclease from *Anabaena*. J Biol Chem.

[B18] Vanden BB (2003). Extremophiles as a source for novel enzymes. Curr Opin Microbiol.

[B19] Blondal T, Thorisdottir A, Unnsteinsdottir U, Hjorleifsdottir S, Aevarsson A, Ernstsson A, Fridjonsson OH, Skirnisdottir S, Wheat JO, Hermannsdottir AG, Sigurdsson ST, Hreggvidsson GO, Smith AV, Kristjansson JK (2005). Isolation and characterization of a thermostable RNA ligase 1 from a Thermus scotoductus bacteriophage TS2126 with good single-stranded DNA ligation properties. Nucleic Acids Res.

[B20] Blondal T, Hjorleifsdottir S, Aevarsson A, Fridjonsson OH, Skirnisdottir S, Wheat JO, Hermannsdottir AG, Hreggvidsson GO, Smith AV, Kristjansson JK (2005). Characterization of a 5'-polynucleotide kinase/3'- phosphatase from bacteriophage RM378. J Biol Chem.

[B21] Welker NE (1967). Purification and Properties of a Thermophilic Bacteriophage Lytic Enzyme. J Virol.

[B22] Desai NA, Shankar V (2000). Purification and characterization of the single-strand-specific and guanylic-acid-preferential deoxyribonuclease activity of the extracellular nuclease from *Basidiobolus haptosporus*. Eur J Biochem.

[B23] Sui MJ, Tsai LC, Hsia KC, Doudeva LG, Chak KF, Yuan HS (2002). Metal ions and phosphate binding in the H-N-H motif: crystal structures of the nuclease domain of ColE7/Im7 in complex with a phosphate ion and different divalent metal ions. Protein Sci.

[B24] Meiss G, Franke I, Gimadutdinow O, Urbanke C, Pingoud A (1998). Biochemical characterization of *Anabaena *sp. strain PCC 7120 non-specific nuclease NucA and its inhibitor NuiA. Eur J Biochem.

[B25] Zhang W, Allen S, Roberts CJ, Soultanas P (2006). The *Bacillus subtilis *Primosomal Protein DnaD Untwists Supercoiled DNA. J Bacteriol.

[B26] Bradford MM (1976). A rapid sensitive method for the quantitation of microgram quantities of protein utilizing the principle of protein-dye binding. Anal Biochem.

[B27] Lineweaver H, Burk D (1934). The determination of enzyme dissociation constants. J Am Chem Soc.

[B28] Hale SP, Poole LB, Gerlt JA (1993). Mechanism of the reaction catalyzed by staphylococcal nuclease: identification of the rate-determining step. Biochemistry.

